# The Chinese version of the world health organization quality of life instrument-older adults module (WHOQOL-OLD): psychometric evaluation

**DOI:** 10.1186/1477-7525-11-156

**Published:** 2013-09-16

**Authors:** Rong Liu, Shaomin Wu, Yuantao Hao, Jing Gu, Jiqian Fang, Nanqiao Cai, Jinxin Zhang

**Affiliations:** 1 School of Public Health, Sun Yat-sen University, 74 Zhongshan RoadII, Guangzhou, 510080, the People’s Republic of China

**Keywords:** Elderly, Quality of life, Reliability, Validity, WHOQOL-OLD

## Abstract

**Background:**

Under the circumstance of global population aging, the issue on how to facilitate the quality of life (QOL) for older people brings us grand challenge. On the way to solve this problem, it is inextricable to measure QOL for older people accurately at onset. This study is aimed at evaluating the reliability and validity of the Chinese version of the World Health Organization Quality of Life Instrument-Older Adults Module (WHOQOL-OLD).

**Methods:**

We received 1005 valid WHOQOL-OLD questionnaires from 1050 respondents who were 60 and older by quota sampling method. To calculate the test-retest correlation coefficient we re-interviewed 101 participants from the community. Psychometric properties were evaluated from the aspect of feasibility, internal consistency reliability, test-retest reliability, content validity, construct validity and discriminant validity.

**Results:**

Missing item responses took up 0.0%-2.7% in the scale. The WHOQOL-OLD showed satisfactory reliability with Cronbach’s Alpha coefficients ranging from 0.711 (Social participation) to 0.842 (Sensory ability) for each domain. The intra-class correlation coefficients (ICC) presenting test-retest reliability were all over 0.7. In Confirmatory Factor Analysis (CFA), Root Mean Square Error of Approximation (RMSEA) was 0.084 (a little more than 0.08) and comparative fit index (CFI) 0.95 (>0.90) which meant acceptable construct validity. There were higher correlation coefficients between items and their hypothesized domains than other domains (P < 0.001), indicating good content validity. The results of t-test showed good discriminant validity of the WHOQOL-OLD between the healthy group and the unhealthy group (P < 0.0083).

**Conclusion:**

The Chinese version of WHOQOL-OLD showed good feasibility, reliability and validity in this study. However, before it can be used national-widely, further research should be conducted in other areas of China.

## Background

The sixth census conducted by the Chinese government indicates that life expectancy of Chinese people has increased from 71.40 years old in 2000 to 74.83 in 2010 [1]. Along with the rising life expectancy and decreasing fertility rate, another inevitable problem comes out—population aging [2]. By 2010, the number of people aged 65 and older in China is about 118.8 million, making up 8.9% of the total population, which exceeds the threshold of 7% for an aging society [3]. On one hand, as they grow old, the seniors have to be faced with chronic disease and organ dysfunction which have negative impact on both their physical and mental health. Under this circumstance, it is necessary to develop a tool to measure the comprehensive health status representing the treatment effect and rehabilitation degree for aged patients and their doctors [4]. On the other hand, more old population and less young proportion will put much more burden on the social support system. Retiring from their job and having few children taking care of them lead them to depend more on pension or medical insurance than they did when they were young. So from the perspective of policy makers and support providers, they need a standard instrument to measure the quality of life for senior citizens to refine their scheme on resource allocation [5]. In order to meet the demand of the policy makers, doctors and the old population themselves, we need to assess quality of life for older people.

Quality of life (QOL) was defined as “individual” perception of their position in life in the context of the culture and value systems in which they live and in relation to their goals, expectations, standards and concerns [6]. The quality of life scale, a new technique generated under the biopsychosocial model [7] is such a kind of instrument to measure one’s health condition and relevant health care need through self- or interviewer-administered questionnaires [8]. Generic instruments and specific ones are two basic approaches to QOL measurements [9]. The former ones mainly include physiological, psychological, social and environmental factors affecting the health of the whole population, while the latter ones are designed for specific population, such as the old [10], the disabled [11] and children [12] etc. The well-developed generic instruments consist of the World Health Organization Quality of Life assessment (WHOQOL-100) [6], the World Health Organization Quality of Life assessment-brief (WHOQOL-BREF) [13], the Medical Outcomes Study 36-item Short Form (SF-36) [14] and so on. For older subjects, the World Health Organization Quality of Life Group (WHOQOL Group) derived the World Health Organization Quality of Life Instrument-Older Adults Module (WHOQOL-OLD) from WHOQOL-BREF by adding specific items important to the seniors such as intimacy, autonomy and death. The process of developing WHOQOL-OLD in Chinese conformed to the existing WHOQOL methodology which included translation of the WHOQOL facet definition and core items, conduct of focus groups, writing national items, development of response scales, construction of pilot instrument, pre-testing and administration of pilot study [15].

In China, as the phenomenon of population aging becomes more and more serious, an increasing number of researchers in medical field are studying the health-related problems occurring under this condition. During these researches, they will confront a common bottleneck: how to measure quality of life in older people precisely? Recently the most often used instruments to complete this assessment are WHOQOL-100, WHOQOL-BREF and SF-36, while the widely-used WHOQOL-OLD around the world is seldom evaluated and used in China.

For the reasons mentioned above, the psychometric properties of the Chinese version of WHOQOL-OLD in a field study will be introduced.

## Methods

### Participants and settings

Our study included 1050 older participants who were 60 and older and living in Guangzhou (known historically as Canton) for more than half a year. The cut point of 60 years was determined according to the Elderly Protection Law of the People’s Republic of China. Then we excluded those who were suffering from senile dementia or reluctant to participate in this study so as to avoid invalid questionnaires. After understanding the objective and importance of this research, all of the subjects were willing to sign on the informed consent form and filled in the questionnaires. This study was approved by the Ethic Committee of School of Public Health, Sun Yat-sen University.

### Instrument

#### Demographic and health-related questionnaires

The demographic information part covered age, gender, marital status, education degree, residence, volunteer activities and occupation. Moreover the health-related part included self-reported health condition and the history of tobacco and alcohol use.

#### The Chinese version of WHOQOL-BREF

This scale comprised 24 facets grouped into 4 domains focusing on the Physical, the Psychological, the Social and the Environmental respectively. Besides, there were two general items about health conditions which would be analyzed independently. Revised by experts in relevant fields, the Chinese version of WHOQOL-BREF had been treated as the standard in China [16]. Each item had five Likert response options which were recoded into 1–5 in score. Higher scores meant better quality of life.

#### WHOQOL-OLD

WHOQOL Group began to invent this elderly-specific scale [17] on the basis of WHOQOL-BREF in 2002. In the first stage of research, focus groups from 22 different WHOQOL centers all over the world put forward 33 items to evaluate QOL for older subjects. Then through pilot study on more than 7400 participants worldwide, both the classical and the modern psychometric analyses, focus groups worked out important facets not mentioned in the first version (for example, intimacy relationship, independent ability and death perceptions). In the second round of field study, about 5566 participants from 20 centers joined in the test. Also after data collection and analysis, concise scale was developed which consists of 24 items divided into 6 groups. These 6 domains are Sensory Abilities (SAB), Autonomy (AUT), Past, Present and Future Activities (PPF), Social Participation (SOP), Death and Dying (DAD) and Intimacy (INT). Responses were rated on a 5-point Likert scale. Higher scores indicated better quality of life. This scale showed good reliability and validity in the assessment of QOL for older participants with multi-language versions [18-21].

As the research center in China, department of medical statistics and epidemiology in Sun Yat-sen University along with 21 other centers from around the world worked together in screening items, conducting pilot study [9]. As for the Chinese version of WHOQOL-OLD, we followed the WHOQOL method in the process of translation and response scales development. The translation process had numerous steps. First we invited a bilingual expert to translate the English version of WHOQOL-OLD into Chinese, and then another bilingual expert to back-translate the former version into English. Aimed at adjusting the first Chinese version and develop a new one, we looked for differences between the first English version and the original one. On the basis of the second Chinese version, the first bilingual expert worked out the second English version which was delivered to University of Edinburgh. Upon receiving comments on this version, we made corresponding changes to finalize the Chinese version of WHOQOL-OLD [22].

### Data collection

Subjects were recruited with the method of quota sampling. The sex ratio and age proportion was determined according to the data of the fifth population census in Guangzhou [23]. Male and female participants were fifty-fifty. The percentages of subjects who were 60 ~ 64, 65 ~ 69, 70 ~ 74, 75 ~ 79, 80 ~ 84, 85 ~ were 30%, 27%, 20%, 12%, 7% and 4% respectively. In this research we chose five urban areas and 2 rural–urban fringe areas as the sampling space. Then we used convenience sampling method to recruit subjects from the community, college for older people, nursing house, general hospital, traditional Chinese medicine hospital in these areas. Repeated interview was conducted among 101 subjects with good compliance sampled from the community and the nursing house after one week of the first interview. The questionnaires were completed in the self-administered or interviewer-administered way. In order to guarantee the quality of the survey, all the interviewers were trained by the project manager.

### Statistical analysis

We input data with Epidata3.0 and analysed it with SPSS17.0. For the continuous variables like age and score, we used mean and standard deviation to describe them when they were normal-distributed. Otherwise, we used median and inter-quartile range instead. For the discrete variable like gender, marital status, education degree, we used relative numbers such as frequency, ratio and proportion to describe them. When it comes to the psychometric properties, we choose the following indices to evaluate the instrument. The feasibility of the WHOQOL-OLD was assessed by analysing the response rate of the scale and the percentage of the missing item response. Internal consistency reliability was measured by the Cronbach’s Alpha Coefficient. Alpha coefficient that was more than 0.70 presented good internal consistency [24]. For the test-retest reliability, or the stability and consistency of the scale, we employed the intra-class correlation coefficient (ICC) to measure. ICC values which were greater than 0.80 indicated good test-retest reliability [23]. Content validity, namely the relationship between items and their hypothesized domain, is assessed by Pearson product correlation. We adopted Confirmatory Factor Analysis (CFA) to evaluate the construct validity. A premeditated six-factor model was performed in this study. Also one-factor model was used for comparison. Main Indexes in CFA included absolute fit index such as the Root Mean Square Error of Approximation (RMSEA) and Goodness of Fit Index (GFI) as well as relative ones such as Comparative Fit Index (CFI), Normed Fit Index (NFI) and Non-Normed Fit Index (NNFI). RMSEA demonstrated the unfit degree of the model thus the smaller the better. Generally, RMSEA that was smaller than 0.05 suggested good fit, 0.05-0.08 fair fit while greater than 0.10 poor fit. To the contrary, GFI, CFI, NFI and NNFI closer to 1 especially greater than the cut-off point of 0.90 indicated good fitness [25]. To show that WHOQOL-OLD can also assess quality of life, convergent validity testing was performed by computing Pearson correlation coefficients of subscale scores and total scores between WHOQOL-OLD and WHOQOL-BREF [26]. Some researchers had proposed that different health condition and age would influence the QOL for the old. So in this survey discriminant validity would be assessed by t-test and Bonferroni Method [15] to test statistical significance in domain scores between the healthy and unhealthy which were divided by subjective perception. Effect sizes were also included by reporting the values of Cohen’s d. The small, medium and large effect sizes were d = 0.20, 0.50 and 0.80 [27].

## Results

### General condition

To evaluate the psychometric characteristics of the WHOQOL-OLD, we enrolled older participants living in Guangzhou for more than half a year from July to November in 2011. We totally gave out 1050 questionnaires and received 1005 finished ones, which meant that the response rate was 95.71%. Because this scale was designed to test the subjective perceptions, it was mainly completed by self-administered method. For those who did not understand the meaning of items or those who had reading disability, investigators assisted them with their completion of questionnaires. After deleting questionnaires containing over 20% missing items, there were 965 questionnaires left, which lead to an efficient ratio of 96.01%. In this survey, the oldest participant was 96 years old while the youngest 60. The mean age was 69.38 (*SD* = 7.44) years for the whole sample. The interviewers married made up 73.1% of the total and the widowed 20.5%. Those who received junior education took the largest part, about 29.9%. The self-administered healthy participants took up 66.8%. Those who never used alcohol accounted for 66.0% while those never used tobacco 78.2%.

### Feasibility

The percentage of missing item response varied from 0.00% to 2.7%. In all the domains, the largest percentage of missing value appeared in Intimacy, 2.0-2.7% in the 21th, 23th, 24th item. For the 22th item in Intimacy, “To what extent do you experience love in your life”, it possessed the lowest percentage of missing value in Intimacy, 1.6% (see Table 1).

**Table 1:** Distribution table of missing item response in WHOQOL-OLD

**Item number**	**WHOQOL-OLD**	
	**Missing item number**	**Missing rate (%)**
1	4	0.40
2	5	0.50
3	2	0.20
4	12	1.30
5	6	0.60
6	9	0.90
7	12	1.30
8	10	1.00
9	10	1.00
10	3	0.30
11	4	0.40
12	16	1.70
13	5	0.50
14	3	0.30
15	3	0.30
16	2	0.20
17	2	0.20
18	9	0.90
19	12	1.20
20	4	0.40
21	26	2.70
22	15	1.60
23	19	2.00
24	25	2.60

### Reliability and validity analysis

#### Internal consistency reliability

The Cronbach’s Alpha Coefficients were used to evaluate the internal consistency reliability of the WHOQOL-OLD. For this scale, it was 0.892. The Cronbach’s Alpha Coefficient of each domain is presented in Table 2.

**Table 2:** Cronbach’s alpha coefficient of each domain

**Domain**	*** α***
Sensory ability (SAB)	0.842
Autonomy (AUT)	0.712
Past, present and future activities (PPF)	0.756
Social participation (SOP)	0.711
Death and dying (DAD)	0.839
Intimacy (INT)	0.817
Total Score	0.892

#### Test-retest reliability

In order to assess the stability of scale, we chose 101 interviewers from community with good compliance and stable health status to retest the questionnaire one week later. The intra-class correlation coefficient between test and retest results in subscale score and total score were all over 0.7, which indicated good test-retest reliability. The ICC values are presented in Table 3.

**Table 3:** Analysis of test-retest reliability for WHOQOL-OLD

**Domain**	**ICC**^**1 **^**value**	**95% confidence interval**	***P***
Sensory ability (SAB)	0.766	0.672-0.836	<0.001
Autonomy (AUT)	0.854	0.790-0.900	<0.001
Past, present and future activities (PPF)	0.906	0.863-0.936	<0.001
Social participation (SOP)	0.860	0.799-0.903	<0.001
Death and dying (DAD)	0.896	0.850-0.929	<0.001
Intimacy (INT)	0.902	0.857-0.934	<0.001
TOTAL SCORE	0.875	0.817-0.916	<0.001

^1^*ICC* Intra-class Correlation Coefficients.

#### Construct validity

Construct validity was tested by using confirmatory factor analysis (CFA) to build a six-factor model according to the original scaling construct. Also for comparison purpose, one-factor model was run to test the model fit. The goodness-of-fit results are demonstrated in Table 4. Its RMSEA value was a little higher than 0.08 and GFI a little lower than 0.90, while CFI, NFI and NNFI are all higher than 0.90, leading to acceptable construct validity.

**Table 4:** Goodness-of-fit results of the WHOQOL-OLD

**Model**	**χ2**	**χ2/df**	**RMSEA**	**NFI**	**NNFI**	**CFI**	**GFI**
Six-factor	1614.95	6.81	0.084	0.94	0.94	0.95	0.86
One-factor	5075.21	20.14	0.141	0.49	0.40	0.50	0.39

*df* degree of freedom, *RMSEA* Root Mean Square Error of Approximation, *NFI* Normed Fit Index, *NNFI* Non-Normed Fit Index, *CFI* Comparative Fit Index, *GFI* Goodness of Fit Index.

#### Content validity

We used simple correlation analysis to evaluate the content validity. The results in Table 5 indicated that the correlation coefficients between items and their hypothesized domains were higher than those with other domains.

**Table 5:** Correlation coefficients between items and their hypothesized domains or total scores

**Item number**	**Sensory ability (SAB)**	**Autonomy (AUT)**	**Past, present and future activities (PPF)**	**Social participation (SOP)**	**Death and dying (DAD)**	**Intimacy (INT)**	**Total score**
1	*0.900*	0.261	0.327	0.306	0.363	0.118	0.583
2	*0.890*	0.297	0.337	0.328	0.364	0.135	0.604
3	0.194	*0.780*	0.381	0.344	0.102	0.264	0.466
4	0.305	*0.733*	0.435	0.364	0.200	0.203	0.511
5	0.208	*0.721*	0.416	0.303	0.136	0.313	0.476
6	0.304	0.161	0.221	0.194	*0.830*	0.099	0.491
7	0.361	0.177	0.227	0.199	*0.858*	0.049	0.507
8	0.308	0.158	0.234	0.191	*0.825*	0.098	0.499
9	0.295	0.135	0.240	0.145	*0.786*	0.092	0.464
10	*0.802*	0.253	0.307	0.309	0.321	0.185	0.548
11	0.273	*0.708*	0.491	0.505	0.125	0.279	0.567
12	0.351	0.454	*0.765*	0.556	0.202	0.236	0.582
13	0.279	0.511	*0.749*	0.429	0.234	0.365	0.596
14	0.260	0.407	0.404	*0.703*	0.092	0.256	0.473
15	0.284	0.435	*0.802*	0.516	0.222	0.289	0.582
16	0.295	0.458	0.575	*0.732*	0.246	0.287	0.604
17	0.354	0.425	0.532	*0.792*	0.208	0.282	0.603
18	0.277	0.255	0.464	*0.723*	0.143	0.236	0.480
19	0.346	0.372	*0.722*	0.514	0.200	0.329	0.582
20	*0.696*	0.322	0.420	0.418	0.200	0.237	0.548
21	0.153	0.307	0.382	0.321	0.077	*0.717*	0.472
22	0.145	0.332	0.309	0.310	0.098	*0.794*	0.496
23	0.170	0.256	0.308	0.256	0.078	*0.855*	0.493
24	0.197	0.276	0.321	0.265	0.104	*0.848*	0.508

Italic values present statistical significance (P<0.001) between items and their hypothesized domains.

#### Convergent validity

The indicators of convergent validity, Pearson correlation coefficients between domain scores of WHOQOL-OLD and those of WHOQOL-BREF ranged from 0.153 to 0.541, all statistically significant at the 0.01 level (2-tailed). Stronger correlations, coefficients varying from 0.399 to 0.643, were found in the comparison of WHOQOL-OLD total scores with WHOQOL-BREF domain scores (see Table 6).

**Table 6:** Pearson correlation coefficients between domain scores in WHOQOL-BREF and WHOQOL-OLD

**WHOQOL-BREF**	**WHOQOL-OLD**						
	**SAB**	**AUT**	**PPF**	**SOP**	**DAD**	**INT**	**Total score**
Physical	0.520*	0.420*	0.452*	0.570*	0.249*	0.243*	0.605*
Psychological	0.437*	0.529*	0.541*	0.535*	0.241*	0.322*	0.643*
Social relationships	0.275*	0.270*	0.384*	0.309*	0.188*	0.153*	0.399*
Environment	0.361*	0.509*	0.523*	0.462*	0.229*	0.319*	0.583*

*correlation is significant at the 0.01 level (2-tailed).

*SAB* Sensory ability, *AUT* Autonomy, *PPF* Past, present and future, *SOP* Social participation, *DAD* Death and dying, *INT* Intimacy.

#### Discriminant validity

For the purpose of comparing the differences of subscale scores and total scores between the healthy group and the unhealthy group which were divided by subjective answer in the demographic information and health-related questionnaire, we used t-test and Bonferroni method. For each domain and total scores, the mean scores of the healthy group were all higher than the unhealthy group. The hypothesis test results showed significant difference of mean scores on all domains except for the Death and Dying (*t* = 1.21, *P* = 0.227). The effect size for all the other domains were small or medium with Cohen’s d ranging from 0.21 (Intimacy) to 0.60 (Social Participation), while effect size for Death and Dying was under the critical value of small (Cohen’s d =0.09) (see Table 7).

**Table 7:** Comparison between scores of healthy group and unhealthy group with the WHOQOL-OLD (Mean ± SD)

**Domain**	**Healthy (*N*** **= 542)**	**Unhealthy (*N*** **= 269)**	***t***	***P***	***Cohen’s d***
Sensory ability (SAB)	3.65 ± 0.74	3.22 ± 0.82	7.99	<0.001*	0.55
Autonomy (AUT)	3.57 ± 0.61	3.40 ± 0.64	4.13	<0.001*	0.27
Past, present and future activities (PPF)	3.54 ± 0.53	3.26 ± 0.60	7.11	<0.001*	0.49
Social participation (SOP)	3.62 ± 0.55	3.28 ± 0.59	8.34	<0.001*	0.60
Death and dying (DAD)	3.77 ± 0.91	3.69 ± 0.93	1.21	0.227	0.09
Intimacy (INT)	3.39 ± 0.80	3.22 ± 0.82	2.83	<0.001*	0.21
TOTAL SCORE	3.59 ± 0.45	3.35 ± 0.50	6.89	<0.001*	

*difference is significant at the 0.05/6 =0.0083 level (2-tailed).

## Discussion

As the number of aging population increases dramatically, the health-related quality of life for older adults attracts more and more public attention [28]. Recently, researchers in China often use generic scales such as WHOQOL-100 [29], WHOQOL-BREF [30], SF-36 [31] to measure QOL for older people. Few researchers report the specific scale WHOQOL-OLD. Being the researching center in China of the WHOQOL group, our department participates in the multi-center study from the very beginning [32]. Then we only apply this scale on the patients with prostatic disease [33] other than the general elderly population. In this research we mainly concentrate on the psychometric characteristic evaluation of the WHOQOL-OLD, so as to find the standardized measurement for the old QOL. Via this standard, we can reflect the health status of older people by their subjective perspectives. As a result, the policy makers or the doctors will know more about the changes in elderly quality of life after their intervention or treatments. Through reforms on social security system, which was proposed and agreed on with the knowledge of this objective statistics, we can have a more harmonious and stable society. In the meanwhile, this research push studies in QOL for Chinese old population to move forward, which fill in gaps in specific instrument for the measurement of elderly QOL.

From the efficient ratio 96.01%, missing item rate 0.0%-2.7%, we could conclude that this investigation in the general population of older people showed good acceptance of the WHOQOL-OLD. Cronbach’s Alpha Coefficients of the six domains and total scores were all over 0.7, which implied good internal consistency. Compared with the results from the WHOQOL Group [10], Brazil [21], Norway [19], Turkey [18], in Chinese version, Cronbach’s Alpha Coefficients of Social participation and Intimacy were smaller. One of the possible reasons maybe that after retirement, Chinese old people are treated as vulnerable population and always taken care of by their children or sent to nursing houses instead of leaving them to social activities [34]. In addition, because of traditional Chinese culture, older people are sensitive to topics about sex and may not express their true feeling when filling in questionnaires [35]. So except for the Social participation and Intimacy, Cronbach’s Alpha Coefficients for other domains were considerably close between Chinese version and foreign ones. As to the test-retest validity, intra-class correlation coefficients of subscale scores and total scores for pre and post survey were all over 0.7 (P < 0.001), which was higher than the results from UK [36]. Lower correlations in UK study were explained by reported life changes in the 4 weeks interval, while our participants stayed in relative stable status during a one week interval. In the construct validity analysis, the results of confirmatory factor analysis for six-factor model that RMSEA was 0.084 (a little higher than 0.08) and CFI 0.95 (>0.9) indicated acceptable construct validity, which was close to the findings of WHOQOL Group [33]. Compared to the one-factor model, the six-factor one showed far better goodness of fit. Significant correlation coefficients implied satisfactory convergence of WHOQOL-OLD total scores on WHOQOL-BREF domains. T-test of subscale scores and total scores between healthy group and unhealthy group showed that except for Death and dying (P > 0.05), there were statistical significance between scores of two groups in other domains, suggesting good discriminant validity. But small to medium Cohen’s d indicated that WHOQOL-OLD was not so efficient when used as criteria for health status [27] although it possessed good discriminant validity to distinguish the healthy from the unhealthy.

Though the psychometric property shown in this study seems to be satisfactory, there still exist limitations in the evaluation of the Chinese version of WHOQOL-OLD. What circumscribes the application of this instrument most is the finite population residing in Canton, the largest city in Southern China. Due to Chinese noticeable north–south gradient in dietary habits [37], psychological status [38], disease incidence [39] and economic development [40], a nation-wide sampling is necessary before generalizing the results. Moreover, morbidity information is not recorded, with which we could analyse whether there is different disease severity between those who finish the questionnaires and those who fail to. Since strict quota sampling compliant with standardized proportion of age and gender is nearly impossible in pragmatic operation, the sample may not appropriately represent the general old population. Therefore, further investigations should be carried out to make up for these limitations.

## Conclusions

As a conclusion, among older people, there exist similar factors affecting their QOL as the general population as well as specific ones only for the old. The generic version of WHOQOL has been well-developed, but it lacks consideration of characteristics in older people. Based on the scale for the general population, we add in important questions relative to older subjects (such as Intimacy, Autonomy, Social participation and Death and dying). This survey demonstrates good feasibility, reliability and validity of the Chinese version of the WHOQOL-OLD. Combined with the generic scale, it can be used to construct an evaluation system for measuring quality of life among older people.

This research is only conducted in the area of Guangzhou. Next we want to increase the sample size and make multi-center research in China to evaluate its psychometric characteristics, which is prepared for the nation-wide application of the Chinese version of the WHOQOL-OLD.

## Consent

Written informed consent was obtained from the subject for the publication of this report and any accompanying images.

## Abbreviations

QOL: Quality of life; WHOQOL Group: The World Health Organization Quality of Life Group; WHOQOL-OLD: The world health organization quality of life instrument-older adults module; WHOQOL-100: World health organization quality of life assessment; WHOQOL-BREF: The world health organization quality of life assessment-brief; SF-36: The medical outcomes study 36-item short form; ICC: Intra-class correlation coefficients; CFA: Confirmatory factor analysis; RMSEA: Root mean square error of approximation; CFI: Comparative fit index; NFI: Normed fit index; NNFI: Non-normed fit index; CFI: Comparative fit index; GFI: Goodness of fit index.; SAB: Sensory abilities; AUT: Autonomy; PPF: Past, present and future activities; SOP: Social participation; DAD: Death and dying; INT: Intimacy.

## Competing interests

The authors declare that they have no competing interests.

## Authors’ contributions

RL conceptualized and designed the study, acquired, analysed and interpreted the data, drafted and revised the manuscript. SW conceptualized and designed the study, acquired, analysed and interpreted the data, and drafted the manuscript. YH conceptualized and designed the study, supervised the data analysis and revised the manuscript. JG conceptualized and designed the study, revised the manuscript, JF conceptualized and designed the study, revised the manuscript, NC acquired, analysed, interpreted the data, and revised the manuscript. JZ conceptualized and designed the study, revised the manuscript. All authors read and approved the final manuscript.
